# Immunogenicity and Reactogenicity of 2009 Influenza A (H1N1) Inactivated Monovalent Non-Adjuvanted Vaccine in Elderly and Immunocompromised Patients

**DOI:** 10.1371/journal.pone.0027214

**Published:** 2011-11-08

**Authors:** João L. Miraglia, Edson Abdala, Paulo M. Hoff, André M. Luiz, Danise S. Oliveira, Carla G. S. Saad, Ieda M. M. Laurindo, Ana T. R. Viso, Angela Tayra, Lígia C. Pierrotti, Luiz S. Azevedo, Lúcia Maria A. Campos, Nádia E. Aikawa, Maria do Carmo S. T. Timenetsky, Expedito Luna, Maria Regina A. Cardoso, José da S. Guedes, Isaias Raw, Jorge Kalil, Alexander R. Precioso

**Affiliations:** 1 Butantan Institute - Clinical Trials Division, São Paulo, Brazil; 2 Instituto do Câncer do Estado de São Paulo (ICESP), Faculdade de Medicina da Universidade de São Paulo, São Paulo, Brazil; 3 Centro de Referência para Imunobiológicos Especiais (CRIE), Hospital das Clínicas da Universidade de São Paulo, São Paulo, Brazil; 4 Division of Rheumatology, Faculdade de Medicina da Universidade de São Paulo, São Paulo, Brazil; 5 Centro de Referência e Treinamento DST/AIDS (CRT-DST/AIDS) of the State of São Paulo, São Paulo, Brazil; 6 Renal Transplantation Unit, Faculdade de Medicina da Universidade de São Paulo, São Paulo, Brazil; 7 Pediatric Rheumatology Unit, Children's Institute, Hospital das Clínicas da Faculdade de Medicina da Universidade de São Paulo, São Paulo, Brazil; 8 Adolfo Lutz Institute, São Paulo, Brazil; 9 Instituto de Medicina Tropical da Universidade de São Paulo, São Paulo, Brazil; 10 Departamento de Epidemiologia da Faculdade de Saúde Pública da Universidade de São Paulo, São Paulo, Brazil; 11 Pediatrics Department, Instituto da Criança do Hospital das Clínicas da Faculdade de Medicina da Universidade de São Paulo, São Paulo, Brazil; University of Hyderabad, India

## Abstract

**Background:**

Immunosuppressed individuals present serious morbidity and mortality from influenza, therefore it is important to understand the safety and immunogenicity of influenza vaccination among them.

**Methods:**

This multicenter cohort study evaluated the immunogenicity and reactogenicity of an inactivated, monovalent, non-adjuvanted pandemic (H1N1) 2009 vaccine among the elderly, HIV-infected, rheumatoid arthritis (RA), cancer, kidney transplant, and juvenile idiopathic arthritis (JIA) patients. Participants were included during routine clinical visits, and vaccinated according to conventional influenza vaccination schedules. Antibody response was measured by the hemagglutination-inhibition assay, before and 21 days after vaccination.

**Results:**

319 patients with cancer, 260 with RA, 256 HIV-infected, 149 elderly individuals, 85 kidney transplant recipients, and 83 with JIA were included.

The proportions of seroprotection, seroconversion, and the geometric mean titer ratios postvaccination were, respectively: 37.6%, 31.8%, and 3.2 among kidney transplant recipients, 61.5%, 53.1%, and 7.5 among RA patients, 63.1%, 55.7%, and 5.7 among the elderly, 59.0%, 54.7%, and 5.9 among HIV-infected patients, 52.4%, 49.2%, and 5.3 among cancer patients, 85.5%, 78.3%, and 16.5 among JIA patients. The vaccine was well tolerated, with no reported severe adverse events.

**Conclusions:**

The vaccine was safe among all groups, with an acceptable immunogenicity among the elderly and JIA patients, however new vaccination strategies should be explored to improve the immune response of immunocompromised adult patients. (ClinicalTrials.gov, NCT01218685)

## Introduction

The novel influenza A (H1N1) virus was first identified in Mexico in March of 2009 [1], and its rapid global spread made the World Health Organization (WHO) declare on June 11, 2009 that a pandemic was under way [2].

According to the WHO, adults and children older than 6 months of age presenting immunosuppressive conditions, and the elderly should be vaccinated against seasonal influenza, since they suffer with serious morbidity and mortality from the disease [3]. These recommendations were extended to the pandemic (H1N1) 2009 virus, and the Brazilian Ministry of Health conducted a nationwide vaccination campaign on March of 2010, vaccinating more than 80 million individuals [4], [5], [6].

As this pandemic virus might circulate as the dominant strain for several years and vaccination will be the most effective morbidity and mortality preventive measure among the immunosuppressed population, to obtain information on the safety and immunogenicity of this vaccine is crucial to improve vaccination strategies among them.

Hence, this study was designed to evaluate the immunogenicity and reactogenicity of an inactivated, split-virus, monovalent, non-adjuvanted 2009 pandemic influenza A (H1N1) vaccine among the elderly, HIV-infected, rheumatoid arthritis (RA), cancer, kidney transplant, and juvenile idiopathic arthritis (JIA) patients.

## Methods

### Study Design

This multicenter prospective observational cohort study was conducted from March, 2010 to July, 2010 at São Paulo, Brazil. Participating clinical sites included the Instituto do Câncer do Estado de São Paulo (ICESP), Renal Transplantation Unit, Division of Rheumatology, Pediatric Rheumatology Unit from Children's Institute, all from Faculdade de Medicina da Universidade de São Paulo, Centro de Referência para Imunobiológicos Especiais (CRIE)/Hospital das Clínicas da Universidade de São Paulo, and Centro de Referência e Treinamento em DST/AIDS (CRT-DST/AIDS) of the State of São Paulo. Patients were invited to participate in the study during their routine clinical visits at one of the sites.

### Ethics Statement

The study was approved by the Ethics Committees of the Faculdade de Medicina da Universidade de São Paulo, ICESP, CRT-DST/AIDS of the State of São Paulo, the Brazilian federal health regulatory agency (ANVISA), and was registered at ClinicalTrials.gov (NCT01218685). The study was conducted in accordance with the principles of the Declaration of Helsinki and Good Clinical Practices [7]. Participants were screened for eligibility, and enrolled by the Principal Investigators following the signature of a written informed consent. Those younger than 18 years of age had the written informed consent signed by a legally acceptable representative.

### Study Population

Children 6 months of age and older with JIA [8], kidney transplant recipients, RA [9], HIV-infected, and cancer patients 18 years of age and older, and elderly 60 years of age and older without any immunosuppressive condition, hereafter referred to as the elderly, were eligible to participate in the study (See Appendix S1 for full eligibility criteria).

### Immunogenicity and safety endpoints

The co-primary immunogenicity endpoints were the proportions of seroprotection (postvaccination hemagglutination-inhibition (HI) antibody titers ≥1∶40), seroconversion (HI antibody titer prevaccination <1∶10 and postvaccination ≥1∶40 or prevaccination ≥1∶10 and an increase by a factor of four or more postvaccination), and the geometric mean ratio of HI antibody titers.

To be licensed, pandemic influenza vaccines must meet all three immunologic endpoints established for seasonal influenza vaccines: proportions of seroprotection >70% or >60%, of seroconversion >40% or >30%, and geometric mean ratio of HI antibody titers >2.5 or 2.0, for adults aged 18–60 years or over 60 years, respectively [10], [11]. Similar requirements applied to adults aged 18–60 years have been proposed to children [12]. Although these endpoints are not applied to immunocompromised individuals, in this study they were used as parameters to evaluate the response to the vaccine.

The secondary safety endpoint comprised solicited local (pain, bruising, redness, and swelling) or systemic (fever, chills, malaise, myalgia, arthralgia, nausea, and headache) adverse events (AEs) reported within the first three days postvaccination. Symptoms were graded as follow: none; mild, if they did not interfere with normal daily activities; moderate, if they interfered with normal daily activities; and severe, if participants could not perform daily activities and/or necessitated medical attention. Fever was defined as an axillary temperature ≥37.8°C.

Prior seasonal influenza vaccination was not evaluated, and a safety monitoring board reviewed reported AEs throughout the study period.

### Study Procedures

The vaccine was administered by intramuscular injection into the deltoid muscle of the nondominant arm. Children aged 6–35 months received two 0.25 mL doses, and children aged 36 months to 8 years received two 0.5 mL doses, both approximately 21 days apart. Children aged 9 years or older, adults, and the elderly received one 0.5 mL dose. Participants, or their representatives, were asked to record local and systemic reactions for the next three days on a diary provided by the investigators.

### Vaccine

The inactivated, split-virus, monovalent, non-adjuvanted 2009 pandemic influenza A (H1N1) vaccine was produced by Butantan Institute/Sanofi Pasteur with seed virus prepared from reassortant vaccine virus A/California/7/2009 (NYMC X-179A). The manufacturing process was identical to that applied for the seasonal vaccine.

The vaccine was supplied as 5 mL multi-dose vials, containing 15 µg of H1 hemagglutinin and 45 µg of thimerosal per 0.5 mL dose, and was stored at 2–8°C until used.

### Laboratory Assays

Blood samples were taken at days 0 and 21 after vaccination or second vaccination for children younger than 9 years of age.

Antibody response was measured by the hemagglutination-inhibition assay according to standard methods at the Adolfo Lutz Institute (São Paulo, Brazil) [13]. Titers were tested at an initial dilution of 1∶10, at a final dilution of 1∶2560, and for the purpose of calculations negative titers had assigned a value of 1∶5. Samples were tested in duplicate, and geometric mean values used in the analyses.

### Statistical Analysis

The proportions of seroprotection, seroconversion, and the geometric mean ratio of HI antibody titers were obtained for each group with their respective 95% confidence intervals (CI). A sensitivity analysis, excluding the participants with prevaccination HI antibody titers ≥1∶40, was also performed with these immunologic parameters. Analyses stratified by age (>60 or ≤60 years) were performed for kidney transplant recipients, RA, HIV-infected, and cancer patients. The distribution of HI antibody titers in each group was described with reverse cumulative distribution curves.

The geometric mean ratio of HI antibody titers and the proportion of seroconversion were compared between participants with prevaccination HI antibody titers <1∶40 and ≥1∶40 in each group by the two-sided Wilcoxon rank-sum test, and two-sided Fisher's exact test, respectively.

Linear and logistic regression were used to evaluated the impact of prevaccination HI titers ≥1∶40 on the geometric mean ratio of HI titers, and on seroconversion, respectively. For the linear regression models, the geometric mean ratios of HI titers were natural log transformed, and the coefficients of the prevaccination HI titers ≥1∶40 variable (coded: 0/no, 1/yes) were exponentiated to obtain the ratio of the geometric mean ratio of HI titers for participants with prevaccination HI titer ≥1∶40 and participants with prevaccination HI titer <1∶40. For linear and logistic regression, the initial full models were adjusted for age and gender, and the variables included in the final regression models were selected by the change-in-estimate procedure with backward elimination [14]. At each stage, the variable for which removal caused the smallest change in the prevaccination HI titers ≥1∶40 variable (coded: 0/no, 1/yes) regression coefficient was removed, given that this change was smaller than 10%. The variances of regression coefficients were obtained by the Huber-White sandwich estimator [15], [16], and Firth's penalized likelihood approach was used to address complete separation [17].

HI antibody titers were natural log transformed for the analyses, and exact (Clopper-Pearson) CIs were calculated for proportional endpoints.

The percentages of local and systemic adverse envents were calculated for each group with their respective 95% confidence intervals (CI).

All statistical tests used a significance level of 0.05, and all analyses were performed by one of the authors (J.L.M.) with Stata 10.1 (StataCorp. 2007. Stata Statistical Software: Release 10. College Station, TX: StataCorp LP).

## Results

### Study Population

The study enrolled 1152 participants, including 319 patients with cancer, 260 with RA, 256 HIV-infected, 149 elderly individuals, 85 kidney transplant recipients, and 83 with JIA.

The baseline characteristics of participants according to study group are described in Table 1. All groups of immunocompromised individuals, besides JIA, included participants older than 60 years of age. More females were included among the elderly, RA, and cancer patients, while more males were included among HIV-infected patients, with no significant difference in the proportion of males and females included among kidney transplant recipients and JIA patients. The proportion of prevaccination HI antibody titers ≥1∶40 ranged from 4.1% among cancer patients to 21.7% among JIA patients.

**Table 1:** Baseline Characteristics of the Study Participants, According to Group.

	Cancer	Rheumatoid Arthritis	HIV–Infected	Elderly	Kidney Transplant	Juvenile Idiopathic Arthritis
	(N = 319)	(N = 258)	(N = 255)	(N = 149)	(N = 85)	(N = 83)
**Age—yr**						
Median	62	58	45	68	50	13
Range	17–90	24–83	22–75	60–89	19–69	3–23
**Sex**						
Female						
%	64.6	85.7	8.6	69.1	47.1	60.2
(95% CI)	(59.1–69.8)	(80.8–89.7)	(5.5–12.8)	(61.0–76.4)	(36.1–58.2)	(48.9–70.8)
Male						
%	35.4	14.3	91.4	30.9	52.9	39.8
(95% CI)	(30.2–40.9)	(10.3–19.2)	(87.2–94.5)	(23.6–39.0)	(41.8–63.9)	(29.2–51.1)
**Prevaccination HI titers ≥1∶40**						
%	4.1	12.3	8.6	12.8	5.9	21.7
(95% CI)	(2.2–6.9)	(8.6–16.9)	(5.5–12.7)	(7.9–19.2)	(1.9–13.2)	(13.4–32.1)

### Immunogenicity

**Figure 1:**
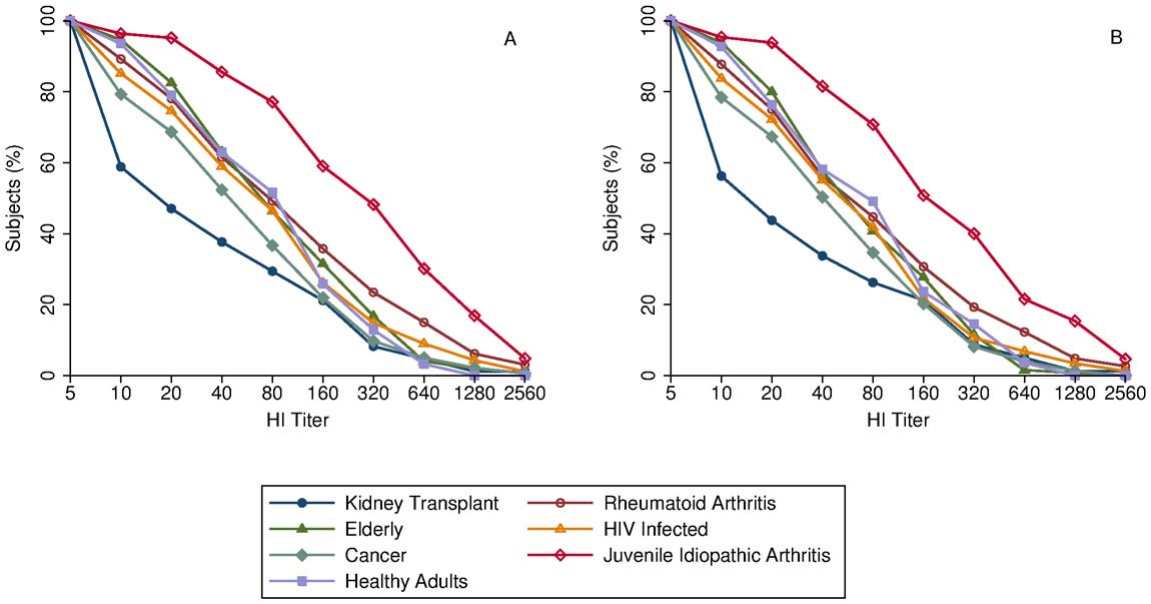
Reverse Cumulative Distribution Curves for Hemagglutination-Inhibition Antibodies Titers on Day 21 After Vaccination. (A) In the entire study population and (B) Excluding participants with prevaccination HI titers ≥1∶40. The limit of detection was a titer of 1∶10. Titers are expressed as the reciprocal of the dilution.

Table 2 describes the antibody responses after vaccination of the entire study population, according to immunosuppressive condition. All study groups achieved a geometric mean ratio of HI antibody titers >2.5, which ranged from 3.2 among kidney transplant recipients to 16.5 among JIA patients. Only kidney transplant recipients did not reach a proportion of seroconversion >40%, while in the remaining groups it ranged from 49.2% among cancer patients to 78.3% among JIA patients. The elderly presented a proportion of seroprotection >60%, and JIA patients >70%, while in the remaining groups it ranged from 37.6% among kidney transplant recipients to 59% among the HIV infected. Figure 1. (A) shows the distribution of HI antibody titers of the entire study population by study group.

**Table 2:** Antibody Responses After Vaccination as Measured with the Hemagglutination–Inhibition Assay, According to Group.

	Cancer	Rheumatoid Arthritis	HIV Infected	Elderly	Kidney Transplant	Juvenile Idiopathic Arthritis
Entire Population	(N = 319)	(N = 260)	(N = 256)	(N = 149)	(N = 85)	(N = 83)
*Baseline*						
Geometric mean titer	6.4	8.3	6.8	9.4	6.8	10.6
(95% CI)	(6.0–6.8)	(7.4–9.2)	(5.8–7.9)	(8.1–10.9)	(5.8–7.9)	(8.1–13.8)
*Postvaccination*						
Geometric mean titer	34	61.8	46.2	53.4	21.3	175.4
(95% CI)	(28.9–40.0)	(50.2–76.1)	(38.1–55.9)	(42.9–66.4)	(15.1–30.2)	(124.6–246.8)
Geometric mean titer ratio	5.3	7.5	5.9	5.7	3.2	16.5
						
(95% CI)	(4.6–6.2)	(6.1–9.1)	(5.0–6.9)	(4.7–6.9)	(2.3–4.4)	(11.8–23.2)
Seroconversion^a^–%	49.2	53.1	54.7	55.7	31.8	78.3
(95% CI)	(43.6–54.8)	(46.8–59.3)	(48.4–60.9)	(47.3–63.8)	(22.1–42.8)	(67.9–86.6)
Seroprotection^b^–%	52.4	61.5	59	63.1	37.6	85.5
(95% CI)	(46.7–57.9)	(55.3–67.5)	(52.7–65.1)	(54.8–70.8)	(27.4–48.8)	(76.1–92.3)

Sensitivity Analysis^c^	(N = 306)	(N = 228)	(N = 234)	(N = 130)	(N = 80)	(N = 65)
*Baseline*						
Geometric mean titer	5.8	6.2	6.5	7.1	5.8	6
(95% CI)	(5.6–6.0)	(5.9–6.6)	(6.1–6.9)	(6.5–7.8)	(5.4–6.3)	(5.4–6.7)
*Postvaccination*						
Geometric mean titer	31.2	50.7	39.2	44	19.7	133.5
(95% CI)	(26.6–36.7)	(40.8–63.0)	(32.3–47.5)	(35.4–54.8)	(13.7–28.2)	(89.9–198.1)
Geometric mean titer ratio	5.4	8.2	6	6.2	3.4	22.3
(95% CI)	(4.6–6.3)	(6.6–10.1)	(5.1–7.2)	(5.0–7.6)	(2.4–4.7)	(15.1–32.8)
Seroconversion^a^–%	48.6	53.9	53.8	56.9	33.8	80
(95% CI)	(42.9–54.4)	(47.2–60.5)	(47.2–60.3)	(48.0–65.6)	(23.2–44.3)	(68.2–88.9)
Seroprotection^b^–%	50.3	56.1	55.1	57.7	33.8	81.5
(95% CI)	(44.6–56.1)	(49.4–62.7)	(48.5–61.6)	(48.7–66.3)	(23.2–44.3)	(70.0–90.1)

Anti–hemagglutinin antigen antibody titers below the detection limit (i.e., <1∶10) were assigned a value of 1∶5 for purposes of calculations.

a HI titer prevaccination <1∶10 and postvaccination ≥1∶40, or prevaccination ≥1∶10 and an increase by a factor of four or more postvaccination.

b HI antibody titer ≥1∶40.

c The sensitivity analysis excluded the participants with prevaccination HI antibody titers ≥1∶40.

The sensitivity analysis (Table 2. and Figure 1. (B)) showed similar results to those observed when the entire study population was analyzed.

The age-stratified analyses only had an impact among kidney transplant recipients, showing that among this population those older than 60 years of age did not achieve any of the three immunologic endpoints evaluated. (Table 1. and Table 2. of the Appendix S1).

### Prevaccination HI Antibody Titers ≥1∶40 and Immune Response

The unadjusted and adjusted analyses showed that participants with prevaccination HI antibody titers ≥1∶40 had statistically significant smaller geometric mean ratios of HI antibody titers, when compared to participants with prevaccination HI antibody titers <1∶40, among the elderly, RA, and JIA patients, with a similar finding among kidney transplant recipients only in the adjusted analyses (Table 3. and Table 4. of the Appendix S1). The linear regression models identified a relative reduction of the geometric mean ratio of HI antibody titers among participants with prevaccination HI titer ≥1∶40, when compared to participants with prevaccination HI titer <1∶40, of 65.8% (95% CI: 47.8–77.6%; *P*<0.0001) among kidney transplant recipients, of 47.4% (95% CI: 11.3–68.8%; *P* = 0.016) among RA patients, of 47.8% (95% CI: 8.4–70.3%; *P* = 0.024) among the elderly, and of 71.8% (95% CI: 47.5–84.9%; *P* = 0.0001) among IJA patients.

In all study groups, there were no significant differences in seroconversion among participants with prevaccination HI titers ≥1∶40 and participants with prevaccination HI titers <1∶40, in both unadjusted and adjusted analyses (Table 3. and Table 5. of the Appendix S1).

### Safety

Local and systemic AEs are described in Table 3. The most frequently reported local AE within 3 days after vaccination among all study groups was pain, which varied from 1.6% among HIV-infected patients to 33.8% among JIA patients. Among systemic AEs, headache and chills were reported by all study groups, while fever was only reported by kidney transplant recipients and RA patients. Most systemic AEs were reported with a frequency of less than 10%, with only RA and JIA patients reporting frequencies greater than 20%. No severe AEs were reported.

**Table 3:** Local and Systemic Adverse Events Within 3 days After Receipt of Vaccination, According to Group.

	Cancer	Rheumaztoid Arthritis	HIV-Infected	Elderly	Kidney Transplant	Juvenile Idiopathic Arthritis
	(N = 305)	(N = 257)	(N = 253)	(N = 106)	(N = 68)	(N = 80)
	*percent*					
	*(95% confidence interval)*					
**Local**						
Pain	6.3	6.6	1.6	4.7	5.8	33.8
	(3.8–9.6)	(3.9–10.4)	(0.4–4.0)	(1.5–10.7)	(1.6–14.4)	(23.6–45.2)
Redness	0	0.8	0.8	2.8	0	1.3
	(0–1.2)	(0.1–2.8)	(0.1–2.8)	(0.6–8.0)	(0–5.3)	(0–6.8)
Swelling	0	2.3	0.8	0	0	0
	(0–1.2)	(1.0–5.0)	(0.1–2.8)	(0–3.4)	(0–5.3)	(0–4.5)
Bruising	0.7	*	0.8	2.8	0	2.5
	(0.1–2.3)		(0.1–2.8)	(0.6–8.0)	(0–5.3)	(0.3–8.7)
**Systemic**						
Fever	0	5.4	0	0	4.4	0
	(0–1.2)	(3.0–9.0)	(0–1.4)	(0–3.4)	(1.0–12.4)	(0–4.5)
Malaise	6.6	a	0.8	8.5	1.5	20
	(4.1–10.0)		(0.1–2.8)	(4.0–15.5)	(0–7.9)	(11.9–30.4)
Nausea	5.6	a	0.4	3.8	0	11.3
	(3.3–8.8)		(0–2.2)	(1.0–9.4)	(0–5.3)	(5.3–20.3)
Headache	12.1	16.3	1.2	8.5	2.9	26.3
	(8.7–16.3)	(12.0–21.4)	(0.2–3.4)	(4.0–15.5)	(0.4–10.2)	(17.0–37.3)
Myalgia	9.5	23.3	0.8	8.5	0	22.5
	(6.5–13.4)	(18.3–29.0)	(0.1–2.8)	(4.0–15.5)	(0–5.3)	(13.9–33.2)
Arthralgia	4.9	16.3	0.4	8.5	0	17.5
	(2.8–8.0)	(12.0–21.4)	(0–2.2)	(4.0–15.5)	(0–5.3)	(9.9–27.6)
Chills	5.9	7.4	0.8	5.7	1.5	8.8
	(3.5–9.2)	(4.5–11.3)	(0.1–2.8)	(2.1–11.9)	(0–7.9)	(3.6–17.2)

a These adverse events were not evaluated among rheumatoid arthritis patients.

## Discussion

Immunosuppressed and elderly individuals present not only high rates of infection with human seasonal influenza virus, but also an increased risk to suffer with severe illness [18], [19], and although available, vaccination against seasonal influenza promotes a diminished immune response among them, when compared to healthy or younger individuals [19], [20].

Monovalent, inactivated, non-adjuvanted pandemic 2009 influenza A (H1N1) vaccines have been evaluated in the general population [21], [22], [23], [24]. Randomized placebo-controlled trials have demonstrated that these vaccines are safe and widely immunogenic, with seroprotection rates among healthy adults, adolescents, and the elderly ranging from 94–98%, 94–97%, and 79–93%, respectively, after a single dose of 15 µg of antigen, and greater than 98% among healthy children, after two doses of 15 µg of antigen. This prospective study found a similar safety profile among immunosuppressed and elderly individuals.

Although in this study the elderly presented a seroprotection rate smaller than previously reported, they showed a satisfactory immune response to the vaccine achieving the three immunologic thresholds established for the licensure of pandemic influenza vaccines (a proportion of seroprotection >60%, of seroconversion >30%, and a geometric mean ratio of HI antibody titers >2.0). In the remaining groups, these parameters varied according to the underlying chronic disease, and it has been demonstrated that they may be diminished when compared to those observed in healthy individuals.

HIV-infected individuals had a diminished immune response to monovalent, inactivated, non-adjuvanted pandemic (H1N1) 2009 vaccines when compared to healthy controls [25], [26]. The proportions of seroprotection and seroconversion observed among them ranged from 50–65% and 39–68%, respectively [26], [27], [28], responses that are in agreement with our findings. Notably, most individuals in these previous studies were taking antiretroviral therapy (82–99%), and presented high median/mean CD4 cell counts at the moment of vaccination (411–581 cell/µl).

A single study evaluated the same vaccine used in our study in RA patients and healthy adults [29], and showed an acceptable and comparable safety profile between them. The proportions of seroprotection and seroconversion were 60.1% and 53.4%, considered significantly different from the proportions observed among healthy individuals, and similar to the proportions of seroprotection and seroconversion of 61.5% and 53.1%, respectively, observed in our study.

No study, that we are aware of, has evaluated the monovalent, inactivated, non-adjuvanted pandemic 2009 influenza A (H1N1) vaccine in adult kidney transplant recipients, cancer, and JIA patients. Data derived from seasonal influenza vaccination indicate that antibody responses are diminished among recipients of solid organ transplants, including kidney transplant, and among cancer patients [19]. Similar diminished humoral responses were found to the vaccine evaluated in our study in these populations, with kidney transplant recipients being the only group that did not reach acceptable proportions of both seroprotection and seroconversion, and individuals older than 60 years of age in this group not reaching acceptable values for any of the three immunologic endpoints evaluated.

In our study the JIA patients were the only ones that showed a proportion of seroprotection >70%, of seroconversion >40%, and a geometric mean ratio of HI antibody titers >2.5, i.e., results similar to those observed in children with rheumatic diseases who received seasonal influenza vaccination [30], [31], [32].

Prevaccination HI antibody titers ≥1∶40 among the study participants reflected the circulation of the pandemic 2009 influenza A (H1N1) virus in Brazil. The higher proportion among JIA patients is in accordance with data showing that children 5–14 years of age had the highest rates of infection with this pandemic virus [33], [34].

We speculated that prevaccination HI antibody titers ≥1∶40 could interfere with overall results and individual immunologic responses, hypotheses that were not confirmed. The sensitivity analysis did not impact any of the three immunologic endpoints across all groups. Furthermore, although most individuals with prevaccination HI antibody titers ≥1∶40 had a smaller increase in the geometric mean ratio of HI antibody titers, than the ones with prevaccination HI antibody titers <1∶40, there were no differences in the proportion of seroconversion between these groups. These findings, along with existing evidence showing that higher levels of antibodies could be associated with higher levels of protection from illness [35], raises the question of whether individuals considered protected could benefit from vaccination as much as those not protected. Further studies addressing this issue are warranted, especially among the population of immunocompromised, since they present an increased risk to suffer with severe illness related to influenza, and therefore would benefit from improved immunity.

Finally, the results from our study should be interpreted with caution, since factors that could have interfered with the immune response to vaccination, including CD4 cell count, HIV viral load, use of immunosuppressive drugs like mycophenolate mofetil or systemic corticosteroids, type of cancer, and timing of vaccination in relation to chemotherapy were not evaluated [19].

In conclusion, this study demonstrated that the pandemic 2009 influenza A (H1N1) monovalent, inactivated, non-adjuvanted vaccine has an acceptable safety profile in the elderly and in the immunocompromised populations herein studied. The immune response observed among the elderly was similar to that observed to seasonal influenza vaccination; JIA patients showed immune responses similar to that observed in health individuals; further studies addressing different vaccination strategies, as multiple doses or adjuvanted-vaccines, among kidney transplant recipients, cancer, HIV-infected, and RA patients are warranted.

## Supporting Information

Appendix S1 (DOC)
