# Supplementary material for: Immunogenicity and Reactogenicity of 2009 Influenza A (H1N1) Inactivated Monovalent Non-Adjuvanted Vaccine in Elderly and Immunocompromised Patients
Source: PLoS One. 2011 Nov 8;6(11):e27214. doi: 10.1371/journal.pone.0027214 (PMC3210772; doi:10.1371/journal.pone.0027214)
Supplement: Appendix S1 — (DOC) [file pone.0027214.s001.doc]

**Appendix S1**

**Eligibility criteria**

**General Inclusion Criteria**

- Aptitude to understand and comply with all procedures required for participation in the study;
- Will to participate and to provide written informed consent.

**General Exclusion Criteria**

- History of egg protein allergy, or allergy to any of the vaccine components;
- History of anaphylaxis or serious reactions to influenza vaccines;
- Vaccination with another inactivated vaccine two weeks prior to enrollment, or vaccination with an attenuated vaccine four weeks prior to enrollment;
- Acute illness and/or fever seven days prior to enrollment;
- Confirmed pandemic influenza H1N1 infection;
- History of progressive neurological disorder, or Guillain-Barrét;
- Participation in another clinical trial during the past six months prior to enrollment;
- Any additional condition that might put a participant at risk or could prevent his/her adequate participation in the study, as evaluated by the principal investigator.

**Specific Inclusion Criteria for HIV-infected individuals**

- Adults 18 years of age and older presenting HIV-1 infection followed-up at the Centro de Referência e Treinamento DST/AIDS of the State of São Paulo.

**Specific Exclusion Criteria for HIV-infected individuals**

- Active neoplastic or infectious disease during the last three months;
- Use of immunomodulators (interferon, interleukins, or systemic corticosteroids in immunosuppressive doses), immunoglobulin, any blood product, or any chemotherapy drug six months prior to enrollment.

**Specific Inclusion Criteria for Patients with Rheumatoid Arthritis**

- Adults 18 years of age and older presenting a diagnosis of rheumatoid arthritis according to the classification criteria of the American College of Rheumatology (ACR).

**Specific Exclusion Criteria for Patients with Rheumatoid Arthritis**

- Patients that have been hospitalized or presenting clinical conditions demanding hospitalization.

**Specific Inclusion Criteria for Patients with Juvenile Idiopathic Arthritis**

- Patients presenting a diagnosis of Juvenile Idiopathic Arthritis according to the classification of the International League of Associations for Rheumatology (ILAR) (persistent and extended pauciarticular-onset, positive or negative rheumatoid factor polyarticular-onset, and systemic-onset).

**Specific Exclusion Criteria for Patients with Juvenile Idiopathic Arthritis**

- Patients that have been hospitalized or presenting clinical conditions demanding hospitalization.

**Specific Inclusion Criteria for Patients with Hematological and Solid Organ Cancers**

- Adults 18 years of age and older presenting hematological and solid organ cancers followed-up at the Cancer Institute of the State of São Paulo (ICESP).

**Specific Exclusion Criteria for Patients with Hematological and Solid Organ Cancers**

- Solid organ or hematopoietic stem cell transplantation;
- HIV infection.

**Specific Inclusion Criteria for Kidney Transplant Recipients**

- Adult kidney transplant recipient, of 18 years of age and older, with 30 days or more from kidney transplant prior to enrollment in the study.

**Specific Exclusion Criteria for Kidney Transplant Recipients**

- Combined kidney-pancreas transplantation;
- HIV infection;
- Cancer;
- Renal allograft loss.

**Specific Inclusion Criteria for Elderly Individuals**

- 60 years of age and older.

**Specific Exclusion Criteria for Elderly Individuals**

- Immunosuppressive condition (cancer, HIV/AIDS, or solid organ or hematopoietic stem cell transplantation);
- Use of immunosuppressive therapy (systemic corticosteroids in a dose ≥ 20mg/day of prednisone, or any other immunosupressive medication).

**Acknowledgments**

**Butantan Institute/Butantan Foundation**

Hernan Chaimovich, Sally M. A. Prado, Ivone Kazuko Yamaguche, Ricardo Oliva, Fábio Alessandro de Freitas, Anatércia Ferreira Bonfim Yano, Maria Aparecida Sakauchi, Célia Sayoko Takata, Fabiana Oliveira Burgos, Luciana Bastos Lima, Maria Amelia Veras, Regina T. S. Ramos.

**Centro de Vigilância Epidemiológica “Prof. Alexandre Vranjac”, Coordenadoria de Controle de Doenças, Secretaria de Estado da Saúde, SP–Divisão de Imunizações**

Helena K. Sato.

**Instituto do Câncer do Estado de São Paulo (ICESP) da Faculdade de Medicina da Universidade de São Paulo**

Adriana Marques da Silva, Adriana Satie Gonçalves Kono Magri, Alex Cintra Ribeiro, Antônia Alice Lima de Souza, Diana Maria Leandro Freire, Doraneide Cheler dos Santos, Fabiana Gonçalves de Aguiar, Gerson Sobrinho Salvador de Oliveira, Isabel Rotter, Joana Pires de Maria, Lígia Camera Pierrotti, Maria Rita da Silva, Maristela Pinheiro Freire, Romilda de Jesus Toledo, Simone de Jesus Crispin Campos, Wania Regina Mollo Baia.

**Centro de Referência para Imunobiológicos Especiais (CRIE) do Hospital das Clínicas da Universidade de São Paulo**

Marta Heloísa Lopes, Ana Marli Christovam Sartori, Karina Takesaki Miyaji, Tânia do Socorro S. Chaves, Fatima Mitie Satake, Elaine Naozuka Simões.

**Centro de Referência e Treinamento em DST/AIDS (CRT-DST/AIDS) of the State of São Paulo**

Ana Cassia dos Reis, Ana Lucia Carvalho Monteiro, Angela Maria Formaggi, Antonio Damião Caetano da Silva, Cristina Langkammer Martins, Daniela Vinhas Bertolini, Denize Lotufo Estevam, Érica Souza, Érika Maria N. Kalmar, Fábio Nogui, Fabio Rodrigues da Silva, Gustavo Mizuno, Ione Aquemi Guibu, José Valdez Ramalho Madruga, Leda Jamal, Ligia Raquel Malheiro de Brito, Luciana Satriano Baptista de Moura, Luiz Martins Jr., Maria Clara Gianna, Maria Crstina Silva, Maria Lucia Mello, Marina Maeda, Marines Aparecida Finotti, Maria do Carmo Brijeiro Oliveira, Maria de Fátima Rodrigues, Maura Regina da Silveira, Nancy de Moraes, Laureano Rodrigues, Rísia Cristina S Oliveira, Rita Manzano Sarti, Roberta Schiavon Nogueira, Rosa Alencar, Sandra Araújo, Suely Kleiman Lewi, Suely Panno, Valdeli Aparecida de Lucca, Suzana Toledo da Silva Leme, Vilciane Borges de Souza, Priscilla de Lima e Menezes, Paula Daher, Celsis de Jesus Pereira.

**Renal Transplantation Unit da Faculdade de Medicina da Universidade de São Paulo**

Cristiano Gamba, Elias David-Neto, Fabiana Agena, Francine Carvalinho Lemos, Juliana  Gerhardt, Maria Cristina Ribeiro de Castro, William Carlos Nahas.

**Pediatric Rheumatoid Unit of Children´s Institute of the Hospital das Clínicas da Faculdade de Medicina da Universidade de São Paulo**

Clovis Silva, Adriana Jesus, Adriana Sallum, Vanessa Guissa e Guilhermes Trudes.

**References**

1. Arnett FC, Edworthy SM, Bloch DA, McShane DJ, Fries JF, et al. (1988) The American Rheumatism Association 1987 revised criteria for the classification of rheumatoid arthritis. Arthritis Rheum 31: 315-324.

2. Petty RE, Southwood TR, Manners P, Baum J, Glass DN, et al. (2004) International League of Associations for Rheumatology classification of juvenile idiopathic arthritis: second revision, Edmonton, 2001. J Rheumatol 31: 390-392

**Tables**

| **Table 1. Antibody Responses After Vaccination as Measured with the Hemagglutination–Inhibition Assay Among Participants ≤ 60 Years of Age, According to Group.** | | | | |
| --- | --- | --- | --- | --- |
|  | **Kidney Transplant** | **Rheumatoid Arthritis** | **HIV–Infected** | **Cancer** |
|  | (N=64) | (N=159) | (N=245) | (N=148) |
| **Baseline** |  |  |  |  |
| Geometric mean titer | 6.8 | 7.3 | 7.8 | 5.8 |
| (95% CI) | (5.7–8.2) | (6.5–8.3) | (7.0–8.6) | (5.4–6.2) |
| HI titer ≥ 1:40 – % | 6.3 | 7.5 | 8.6 | 2.7 |
| (95% CI) | (1.7–15.2) | (4.0–12.8) | (5.4–12.8) | (0.7–6.8) |
| **Postvaccination** |  |  |  |  |
| Geometric mean titer | 25.4 | 69 | 46.6 | 37.8 |
| (95% CI) | (16.8–38.4) | (53.3–89.3) | (38.2–56.8) | (30.0–47.6) |
| Geometric mean  titer ratio | 3.7 | 9.4 | 6.0 | 6.5 |
| (95% CI) | (2.5–5.4) | (7.3–12.2) | (5.1–7.1) | (5.3–8.1) |
| Seroconversion^*^ – % | 37.5 | 57.9 | 55.1 | 56.1 |
| (95% CI) | (25.7–50.5) | (49.8–65.6) | (48.6–61.4) | (47.7–64.2) |
| Seroprotection^†^ – % | 43.8 | 63.5 | 58.8 | 56.8 |
| (95% CI) | (31.4–56.7) | (55.5–71.0) | (52.3–65.0) | (48.4–64.9) |
| Anti–hemagglutinin antigen antibody titers below the detection limit (i.e., <1:10) were assigned a value of 1:5 for purposes of calculations. | | | | |
| ^*^HI titer prevaccination ≤1:10 and postvaccination ≥1:40, or prevaccination ≥1:10 and an increase by a factor of four or more postvaccination.  ^†^HI antibody titer ≥ 1:40. | | | | |

| **Table 2. Antibody Responses After Vaccination as Measured with the Hemagglutination–Inhibition Assay Among Participants > 60 Years of Age, According to Group.** | | | | |
| --- | --- | --- | --- | --- |
|  | **Kidney Transplant** | **Rheumatoid Arthritis** | **HIV–Infected** | **Cancer** |
|  | **(N=21)** | **(N=101)** | **(N=10)** | **(N=171)** |
| **Baseline** |  |  |  |  |
| Geometric mean titer | 6.5 | 10.1 | 11.5 | 6.9 |
| (95% CI) | (4.8–8.9) | (8.2–12.3) | (4.5–29.1) | (6.2–7.7) |
| HI titer ≥ 1:40 – % | 4.8 | 19.8 | 10.0 | 5.3 |
| (95% CI) | (0.1–23.8) | (12.5–28.9) | (0.3–44.5) | (2.4–9.8) |
| **Postvaccination** |  |  |  |  |
| Geometric mean titer | 12.6 | 51.9 | 37.3 | 31.0 |
| (95% CI) | (6.7–23.6) | (36.5–73.9) | (15.3–90.7) | (24.6–39.0) |
| Geometric mean  titer ratio | 1.9 | 5.2 | 3.2 | 4.5 |
| (95% CI) | (1.1–3.6) | (3.8–6.9) | (1.8–5.8) | (3.6–5.5) |
| Seroconversion* – % | 14.3 | 45.5 | 40.0 | 43.3 |
| (95% CI) | (3.0–36.3) | (35.6–55.8) | (12.2–73.8) | (35.7–51.1) |
| Seroprotection^†^ – % | 19.0 | 58.4 | 60.0 | 48.5 |
| (95% CI) | (5.4–41.9) | (48.2–68.1) | (26.2–87.8) | (40.8–56.3) |
| Anti–hemagglutinin antigen antibody titers below the detection limit (i.e., <1:10) were assigned a value of 1:5 for purposes of calculations. | | | | |
| *HI titer prevaccination ≤1:10 and postvaccination ≥1:40, or prevaccination ≥1:10 and an increase by a factor of four or more postvaccination.  ^†^HI antibody titer ≥ 1:40. | | | | |

| **Table 3. Comparison of Geometric Mean Titer Ratio and Percentage of Seroconversion According to Prevaccination HI Antibody Titers.** | | | | | | |
| --- | --- | --- | --- | --- | --- | --- |
|  | **Geometric mean titer ratio** | | ***P*–value^†^** | **Seroconversion* – %** | | ***P*–value^‡^** |
|  | (95% CI) | |  | (95% CI) | |  |
|  | Titer < 1:40 | Titer ≥ 1:40 |  | Titer < 1:40 | Titer ≥ 1:40 |  |
| **Kidney Transplant** | 3.4 | 1.1 | 0.12 | 33.8 | 0 | 0.17 |
|  | (2.4–4.7) | (0.8–1.7) |  | (23.2–44.3) | (0.0–52.2) |  |
| **Rheumatoid Arthritis** | 8.2 | 3.9 | 0.012 | 53.9 | 46.9 | 0.46 |
|  | (6.6–10.1) | (2.4–6.3) |  | (47.2–60.5) | (29.1–65.3) |  |
| **Elderly** | 6.2 | 3.2 | 0.021 | 56.9 | 47.4 | 0.47 |
|  | (5.0–7.6) | (1.8–5.7) |  | (48.0–65.6) | (24.4–71.1) |  |
| **HIV Infected** | 6.0 | 4.3 | 0.34 | 53.8 | 63.6 | 0.50 |
|  | (5.1–7.2) | (2.8–6.6) |  | (47.2–60.3) | (40.7–82.8) |  |
| **Cancer** | 5.4 | 4 | 0.54 | 48.6 | 61.5 | 0.41 |
|  | (4.6–6.3) | (2.2–7.4) |  | (42.9–54.4) | (31.6–86.1) |  |
| **Juvenile Idiopathic Arthritis** | 22.3 | 5.7 | <0.001 | 80 | 72.2 | 0.34 |
|  | (15.1–32.8) | (3.6–9.0) |  | (68.2–88.9) | (49.3–95.1) |  |
| *HI antibody titer prevaccination ≤1:10 and postvaccination ≥1:40, or prevaccination ≥1:10 and an increase by a factor of four or more postvaccination. | | | | | | |
| ^†^Two-sided Wilcoxon rank sum test. | | |  |  |  |  |
| ^‡^Tow-sided Fisher's exaxt test. | |  |  |  |  |  |

| **Table 4.** Final linear regression models evaluating the impact of prevaccination HI titers ≥ 1:40 on the geometric mean increase of HI titers. | | | |
| --- | --- | --- | --- |
|  | Coefficient | *P*-value | 95% CI |
| **HIV-infected*** | -0.40 | 0.078 | (0.04 – -0.85) |
| **Kidney Transplant** | -1.07 | <0.0001 | (-0.65 – -1.50) |
| **Rheumatoid Arthritis^†^** | -0.64 | 0.016 | (-0.12 – -1.17) |
| **Elderly** | -0.65 | 0.024 | (-0.09 – -1.21) |
| **Cancer^‡^** | -0.16 | 0.57 | (0.39 – -0.70) |
| **Juvenile Idiopathic Arthritis** | -1.27 | 0.0001 | (-0.64 – -1.89) |
| *Adjusted for gender. |  |  |  |
| ^†^Adjusted for age. |  |  |  |
| ^‡^Adjusted for gender and age. | |  |  |
|  | |  |  |

| **Table 5.** Final logistic regression models evaluating the impact of prevaccination HI titers < 1:40 on seroconversion. | | | |
| --- | --- | --- | --- |
|  | OR | *P*-value | 95% CI |
| **HIV-infected*** | 0.70 | 0.439 | (0.28–1.74) |
| **Kidney Transplant** | 5.65 | 0.247 | (0.30–106.03) |
| **Rheumatoid Arthritis^†^** | 1.22 | 0.607 | (0.57–2.64) |
| **Elderly^†^** | 1.42 | 0.497 | (0.52–3.86) |
| **Cancer^‡^** | 0.47 | 0.23 | (0.14–1.60) |
| **Juvenile Idiopathic Arthritis*** | 0.74 | 0.634 | (0.21–2.57) |
| *Adjusted for gender. |  |  |  |
| ^†^Adjusted for age. |  |  |  |
| ^‡^Adjusted for gender and age. | |  |  |
